# Co-inoculation of rhizobacteria promotes growth, yield, and nutrient contents in soybean and improves soil enzymes and nutrients under drought conditions

**DOI:** 10.1038/s41598-021-01337-9

**Published:** 2021-11-11

**Authors:** Dilfuza Jabborova, Annapurna Kannepalli, Kakhramon Davranov, Abdujalil Narimanov, Yuriy Enakiev, Asad Syed, Abdallah M. Elgorban, Ali H. Bahkali, Stephan Wirth, R. Z. Sayyed, Abdul Gafur

**Affiliations:** 1 Institute of Genetics and Plant Experimental Biology, Uzbekistan Academy of Sciences, Tashkent Region, 111208 Kibray, Uzbekistan; 2 Division of Microbiology, ICAR-Indian Agricultural Research Institute, Pusa, New Delhi, 110012 India; 3 Leibniz Centre for Agricultural Landscape Research (ZALF), 15374 Müncheberg, Germany; 4 Institute of Microbiology, Academy of Sciences of Uzbekistan, 100128 Tashkent, Uzbekistan; 5 Agro-Technology and Plant Protection. 7, Nikola Pushkarov Institute of Soil Science, Shosse Bankya str., 1331 Sofia, Bulgaria; 6 Department of Botany and Microbiology, College of Science, King Saud University, P.O. Box 2455, Riyadh, 11451 Saudi Arabia; 7 Department of Microbiology, PSGVP Mandal’s, Arts, Science & Commerce College, Shahada, Maharashtra 425409 India; 8 Sinarmas Forestry Corporate Research and Development, Perawang, 28772 Indonesia

**Keywords:** Microbiology, Plant sciences

## Abstract

Drought stress is the major abiotic factor limiting crop production. Co-inoculating crops with nitrogen fixing bacteria and plant growth-promoting rhizobacteria (PGPR) improves plant growth and increases drought tolerance in arid or semiarid areas. Soybean is a major source of high-quality protein and oil for humans. It is susceptible to drought stress conditions. The co-inoculation of drought-stressed soybean with nodulating rhizobia and root-colonizing, PGPR improves the root and the shoot growth, formation of nodules, and nitrogen fixation capacity in soybean. The present study was aimed to observe if the co-inoculation of soybean (*Glycine max* L. (Merr.) nodulating with *Bradyrhizobium japonicum* USDA110 and PGPR *Pseudomonas putida* NUU8 can enhance drought tolerance, nodulation, plant growth, and nutrient uptake under drought conditions. The results of the study showed that co-inoculation with *B. japonicum* USDA110 and *P. putida* NUU8 gave more benefits in nodulation and growth of soybean compared to plants inoculated with *B. japonicum* USDA110 alone and uninoculated control. Under drought conditions, co-inoculation of *B. japonicum* USDA 110 and *P. putida* NUU8 significantly enhanced the root length by 56%, shoot length by 33%, root dry weight by 47%, shoot dry weight by 48%, and nodule number 17% compared to the control under drought-stressed. Co-inoculation with *B. japonicum*, USDA 110 and *P. putida* NUU8 significantly enhanced plant and soil nutrients and soil enzymes compared to control under normal and drought stress conditions. The synergistic use of *B. japonicum* USDA110 and *P. putida* NUU8 improves plant growth and nodulation of soybean under drought stress conditions. The results suggested that these strains could be used to formulate a consortium of biofertilizers for sustainable production of soybean under drought-stressed field conditions.

## Introduction

Soybean (*Glycine max* L.) is a principal oilseed crop grown throughout the world, accounting for about 200 million tons/annum. It is the major source of vegetable oil and vegetable protein food for humans and a high-quality animal feed. About 50–60% more soybean production will be needed by 2050^1^. This global crop is affected by several biotic and abiotic stresses. Drought is one of the crucial abiotic stresses that affect the growth and yield of soybean and many other crops in dry and semiarid regions^2^. Drought stress causes a series of adverse effects on germination, plant growth, development, and yield of different crops, including soybean^3^. Drought negatively impacts the seed germination rate^4^, leaf area^5,6^, flowers^7^, pods^8^, seed^9^, and yield of soybean^10^.

Physiological properties and availability of plant nutrients play essential roles in plant growth, plant development, and yield^11,12^. Drought impacts adverse effects on plant physiological properties such as chlorophyll content^13^, photosynthesis rate^14^, stomatal conductance^15,16^, and transpiration rate^17–19^. Furthermore, water stress significantly reduced plants' nitrogen (N) content^20,21^.

Drought stress affects soil nutrient availability, microbiological parameters, nutrient adsorption, and soil enzyme such as protease, acid, and alkaline phosphomonoesterase^22^. These enzymes mediate protein and phosphate (P) hydrolysis into bioavailable amino acids, organic nitrogen, and soluble P^22^. However, the activities of these enzymes are governed by many factors, such as soil properties, soil organic matter contents, and the availability of organic compounds^23^. This warrants the need to search for sustainable strategies to manage drought stress. Among the various approaches, inoculation of plant growth-promoting rhizobacteria (PGPR) in the rhizosphere of crops has been seen as one of the most suitable strategies to promote plant growth under drought stress conditions^24^. PGPRs are known for their beneficial effects on plant growth, development^25,26^, nutrient uptake^27,28^, and yield under drought stress conditions^24,29^. These inoculants in single or co-inoculation form enhance nodulation^30,31^, nodule weight^30^, nitrogen fixation^32^, plant biomass^33,34^, dry matter and grain yield^35,36^. Moreover, PGPR also help in controlling phytopathogens^37–40^. However, co-inoculation is more effective than single inoculum^31,35,36^. Thus there is a need to search for a good combination of co-inoculations for good growth and yield in soybean. The present study was aimed to evaluate the effect of co-inoculation with *B. japonicum*, USDA 110 and *P. putida* NUU8 significantly enhanced plant and soil nutrients and soil enzymes compared to control under normal and drought stress conditions.

## Methods

### Bacterial culture, soybean seeds, and soil

Bacterial cultures, namely *B. japonicum* USDA 110 and *P. putida* NUU8, were obtained from the culture repository of the Microbiology and Biotechnology Department of the National University of Uzbekistan Tashkent, Uzbekistan. The soybean seeds (*Glycine max* L. Merr.) were collected from Leibniz Centre for Agricultural Landscape Research (ZALF), Müncheberg, Germany. The soil for pot assay was collected from Leibniz Centre for Agricultural Landscape Research (ZALF), Müncheberg, Germany.

### Preparation of *B. japonicum* USDA 110 and *P. putida* NUU8 inoculum

For inoculum preparation, *B. japonicum* USDA110 and *P. putida* NUU8 were grown in Yeast extract mannitol broth and nutrient broth respectively at 30 °C and 120 rpm for 48 h. This inoculum was used for seed bacterization.

### Surface sterilization, germination, and bacterization of seeds

Soybean seeds were surface sterilized in 10% sodium hypochlorite solution for 5 min, followed by three washings with sterile distilled water. Sterilized seeds were germinated in Petri dishes (85 mm × 15 mm). For bacterization, germinated seeds were immersed in culture broth (5 × 10^6^ CFU g^−1^) *B. japonicum* USDA 110 and *P. putida* NUU8 for 10 min, air-dried, and then planted in 1. Kg capacity plastic pots containing 400 g sandy loamy soil.

### Experimental design

The effect of rhizobacteria on soybean growth was studied in pot experiments in a greenhouse at ZALF, Müncheberg, Germany, during July 2019. The experiments were carried out. All the experiments were carried out in a randomized block design with five replications. Experimental treatments included:

- T1-Control under normal water conditions
- T2-Control under drought stress conditions
- T3-Inoculation with *B. japonicum* USDA 110 under normal water conditions
- T4-Inoculation with *B. japonicum* USDA 110 drought stress conditions
- T5-Inoculation with *P. putida NUU8* under normal water conditions
- T6-Inoculation with *P. putida NUU8* under drought stress conditions
- T7-Co-inoculation with *B. japonicum* USDA 110 and *P. putida* NUU8 strains under normal water conditions
- T8-Co-inoculation with *B. japonicum* USDA 110 and *P. putida* NUU8 strains under drought stress conditions.

Plants were grown in pots under greenhouse conditions at 24 °C during the day and 16 °C at night for 30 days. Normal water conditions (70% of the pot capacity) and drought stress conditions (40% of pot capacity) were maintained.

### Measurement of plant growth parameters and plant nutrients

Soybean plants were harvested from pots after 30 days of germination. The measurement of seed germination rate (%), root length (cm), shoot length (cm), root dry weight (mg/g), shoot dry weight (mg/g), and the number of nodules per plant was measured.

For the estimation of plant nutrients, such as nitrogen, phosphorus, potassium, magnesium, sodium, and calcium, 1 g of crushed plant tissue was added in phosphate buffer (pH 6.7), and these nutrients were measured spectrophotometrically (iCAP 6300 Duo, Thermo Fischer Scientific Inc., Waltham, MA, USA)^41,42^. The nitrogen and phosphorus contents of root and shoot were determined from dried plant biomass. For nitrogen estimation, one g of dried leaf biomass was digested in 10 mL concentrated H_2_SO_4_ and 5.0 g catalyst mixture in a digestion tube. The digested and cooled mixture distillate and the distillate was titrated with H_2_SO_4_. The mixture that did not contain leaf biomass served as a control. Total nitrogen was calculated from the blank and sample titer reading^43^.

The P content of plant biomass was first extracted with 0.5 N NaHCO_3_ buffer (pH 8.5) followed by treatment with ascorbic acid. The intensity of the blue color produced was measured at 540 nm. The amount of P from plant biomass was calculated from the standard curve of P^44^. For the estimation of potassium content of the plant, 5 g of the plant biomass was added in 25 mL of ammonium acetate, shaken for 5 min, and filtered. The amount of potassium from the filtrate was measured according to Upadhyay and Sahu^45^. To estimate Na, Mg, and Ca, one g of plant extract was mixed with 80 mL of 0.5 N HCl and incubated for 5 min at 25 °C and filtered. The amount of Na, Mg, and Ca from the filtrate was estimated according to Sahawat^46^.

### Analysis of soil nutrient

The root soil (10 g) of each experimental pot was air-dried and shaken with 100 mL ammonium acetate buffer (0.5 M) for 30 min to displace the adhered nutrients and minerals. Soil organic carbon (SOC), nitrogen, phosphorus, and potassium contents of soil were determined according to the method of Sims^34^. This method mixed 1.0 g of soil with 10 mL of 1 N K_2_Cr_2_O_7_ and 20 mL of concentrated H_2_SO_4_. This suspension was mixed thoroughly and diluted to 200 mL of distilled water, followed by the addition of 10 mL each of H_3_PO_4_ and sodium fluoride. The resulting solution was used to estimate N, P, and K^47^. Blank (without soil) served as control.

### Estimation of soil enzymes

The acid and alkaline phosphomonoesterase activities of soil were assayed according to the method of Tabatabai and Bremner^48^. For this, 0.5 g of moist soil was mixed in 2 mL of modified universal buffer (pH 6.5 for the acid phosphatase and pH 11 for the alkaline phosphatase) and 0.5 mL of p-nitrophenyl phosphate (PNP) substrate solution (0.05 M). The change in the color of solution due to p-nitrophenol (p-NP) production due to acid and alkaline phosphomonoesterase activities was measured at 400 nm, and the amount of p-NP was calculated from a p-NP calibration curve. Solution without soil served as the control. One unit of phosphomonoesterase activity was defined as the amount of enzyme required to liberate 1 mM of p-NP (product) from 1 kg of dried soil at 37 °C per 1 h^48^.

Protease activity was assayed according to the method of Ladd and Butler^49^. For this, 0.5 g of soil was added in 2.5 mL of 0.2 M phosphate buffer (pH of 7.0) and 0.5 mL of 0.03 M N-benzoyl-l-arginine amide (BAA) substrate solution. The amount of ammonium released during the reaction was measured at 690 nm. One unit of protease activity was defined as the amount of enzyme ammonium equivalents released from BAA per minute.

### Statistical analyses

All the experiments were performed in five replicates, and the mean values of five replicates were considered. The data were statistically analyzed by one-way analysis of variance (ANOVA) and multiple comparisons of HSD employing the Tukey test with Stat View Software (SAS Institute, Cary, NC, USA, 1998). The significance of the effect of various treatments on plant growth parameters, plant nutrients, and soil nutrients was determined by the magnitude of the *p*-value (*p* < 0.05 < 0.001).

## Results

### Measurement of plant growth parameters

**Figure 1:**
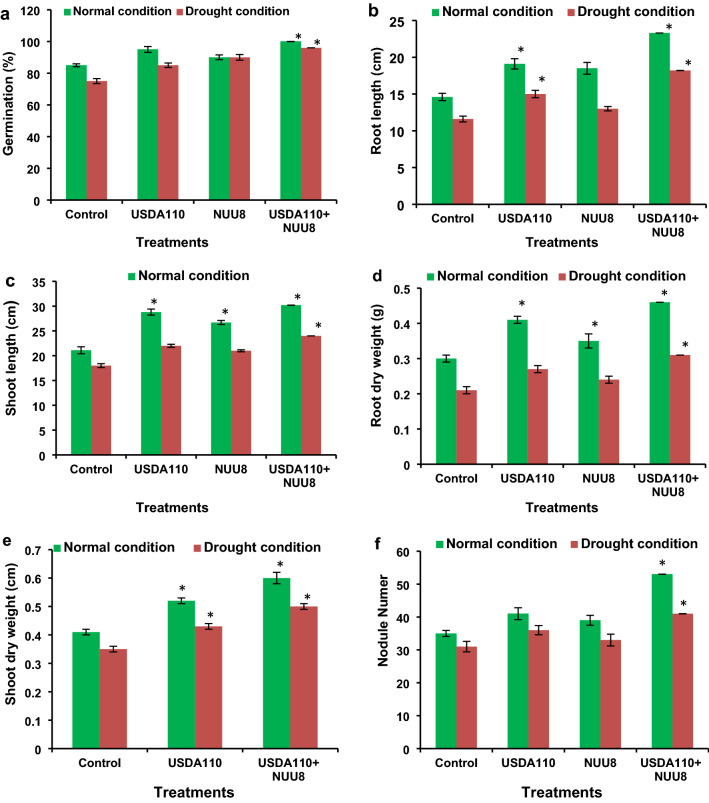
Effect of single inoculation of *B. japonicum* USDA 110, and *P. putida* NUU8 and co-inoculation of *B. japonicum* USDA 110, and *P. putida* NUU8 strains: (**a**) germination, (**b**) root length, (**c**) shoot length, (**d**) root dry weight of soybean under normal and drought conditions, (**e**) shoot dry weight, and (**f**) nodule number per plant. Data are presented as mean + standard deviation of five replicates. *Significant differences (*p* ≤ 0.05).

Drought stress conditions affected the seed germination in soybean (Fig. 1) compared to the normal water conditions. Application of rhizobacteria enhanced seed germination under drought conditions and normal conditions compared to control under drought and normal conditions, respectively. Inoculation of *B. japonicum* USDA 110 alone increased the seed germination by 12.5% under drought conditions and by 10.0% under normal conditions over the control. Co-inoculation of *B. japonicum* USDA 110 and *P. putida* strain NUU 8 significantly improved the seed germination under drought stress and normal water conditions. Under drought conditions, co-inoculation of *B. japonicum* USDA 110 and *P. putida* strain NUU 8 enhanced the seed germination by 16.2% and 13% under drought and normal conditions compared to the control under drought and normal conditions, respectively (Fig. 1a).

The rhizobacterial inoculation significantly improved the growth of the soybean plant under normal and drought stress conditions. Inoculation of *B. japonicum* USDA 110 alone significantly enhanced the root length by 30% (Fig. 1b), shoot length by 36% (Fig. 1c), root dry weight by 33% (Fig. 1d), and shoot dry weight by 26% (Fig. 1e), as compared to the control under normal water conditions. Inoculation of *B. japonicum* USDA 110 under drought stress conditions significantly increased the root length by 29% (Fig. 1b), shoot length by 22% (Fig. 1c), root dry weight by 28% (Fig. 1d), and shoot dry weight by 22% (Fig. 1e) over the control under drought and normal conditions, respectively. Whereas the co-inoculation with *B. japonicum* USDA 110 and *P. putida* strains NUU8 significantly increased the root length, shoot length, root dry weight, shoot dry weight, and nodule number compared to the control under normal and drought conditions.

A 59% and 56% increase in root length (Fig. 1b), 43% and 33% increase in shoot length (Fig. 1c), 53% and 47% rise in root dry weight (Fig. 1d), 48% and 46% improvement in shoot dry weight (Fig. 1e) and 29% and 27% rise in nodule number (Fig. 1f) was evident over the control under normal condition and drought stress, respectively.

### Measurement of plant nutrient contents

Analysis of nutrients in a soybean plant revealed that single inoculation of *B. japonicum* USDA 110 significantly increased N content by 29% and 28%, P content by 15% and 12%, K content by 32 and 28%, Mg content by 12%, and 9.0%, Na content by 50% and 43% and Ca content by 13% and 11% respectively as compared to control under normal condition and drought stress conditions respectively. A single inoculation of *P. putida* NUU8 also exhibited a substantial increase in the nutrient contents. It increases nitrogen by 21% and 17%, P content by 14% and 11%, K content by 30 and 26%, Mg content by 10% and 8.0%, Na content by 45% and 38%, and Ca content by 10% and 8.0% respectively as compared to control under normal condition and drought stress conditions respectively. However, co-inoculation of *B. japonicum* USDA 110 and *P.putida* NNU8 resulted in a significant improvement in N content by 44%, and 35% P content by 34% and 31%, K content by 41% and 28, Mg content by 19% and 15%, Na content by 83% and 50% and Ca content by 41% and 35% over the control under normal condition and drought stress conditions respectively (Table 1).

**Table 1:** Effect of coinoculation with *B. japonicum* USDA 110 and *P. putida* NUU8 and single inoculation *B. japonicum* USDA 110 strains on plant nutrients under normal and drought conditions.

Conditions	Treatments	N (%)	P (%)	K (%)	Mg (%)	Na (%)	Ca (%)
Normal	Control	1.99 + 0.02	0.24 + 0.02	1.45 + 0.01	0.58 + 0.01	0.18 + 0.02	1.20 + 0.01
	USDA 110	2.57 + 0.01*	0.29 + 0.01	1.62 + 0.02	0.61 + 0.01	0.27 + 0.01*	1.22 + 0.01
	NUU8	2.37 + 0.01*	0.21 + 0.01	1.43 + 0.02	0.52 + 0.01	0.21 + 0.01*	1.01 + 0.01
	USDA + NUU8	2.87 + 0.01*	0.33 + 0.01*	2.05 + 0.02*	0.69 + 0.01*	0.33 + 0.03*	1.69 + 0.01
Drought	Control	1.76 + 0.01	0.20 + 0.01	1.34 + 0.01	0.54 + 0.01	0.04 + 0.01	1.01 + 0.01
	USDA 110	2.26 + 0.02*	0.25 + 0.01	1.55* + 0.01	0.56 + 0.01	0.05 + 0.02	1.15 + 0.01
	NUU8	2.01 + 0.02*	0.18 + 0.01	1.21* + 0.01	0.45 + 0.01	0.05 + 0.02	1.01 + 0.01
	USDA + NUU8	2.38 + 0.02*	0.29 + 0.02	1.71 + 0.02*	0.58 + 0.02	0.06 + 0.02*	1.53 + 0.02*

Values are the average of three replicates ± values are standard deviations. Plant nutrient contents were measured after 30 days of plant growth under greenhouse conditions.

*Values significant at *p* 0.01.

### Analysis of soil nutrient contents

Analysis of soil nutrient contents revealed significant improvement in soil N, P, and K content due to rhizobacterial inoculation compared to control (Table 2). Inoculation with *B. japonicum* USDA 110 alone significantly increased total N content by 16% and 12%, P content by 18% and 16%, and K content by 16% and 14%, respectively, compared to the control under normal conditions and drought conditions respectively. In comparison, single inoculation with *P. Putida* NUU8 increased total N content by 13% and 11%, P content by 16% and 13%, and K content by 13% and 11% compared to the control under normal conditions and drought conditions, respectively. However, the highest N, P, and K values were observed in soil amended co-inoculation with *B. japonicum* USDA 110 and *P. putida* NUU8 treatment under normal and drought stress conditions. The co-inoculation significantly increased the total N content by 20% and 23%, P content by 14% and 12%, and K content by 48% and 30%, respectively, compared to the control under conditions and drought stress conditions, respectively (Table 2).

**Table 2:** Effect of coinoculation with *B. japonicum* USDA 110 and *P. putida* NUU8 and single inoculation *B. japonicum* USDA 110 strains on soil nutrients under normal and drought conditions.

Conditions	Treatments	Total N (%)	P (mg)	K (mg)
Normal	Control	0.080 ± 0.01	4.61 ± 0.02	4.25 ± 0.02
	USDA 110	0.093 ± 0.03*	4.84 ± 0.02	4.88 ± 0.03*
	NUU8	0.087 ± 0.03*	4.11 ± 0.02	4.13 ± 0.03*
	USDA + NUU8	0.096 ± 0.02*	5.24 ± 0.02*	6.30 ± 0.03*
Drought	Control	0.075 ± 0.02	4.02 ± 0.01	3.66 ± 0.01
	USDA 110	0.084 ± 0.01*	4.50 ± 0.01	4.20 ± 0.02*
	NUU8	0.077 ± 0.01*	4.19 ± 0.01	4.16 ± 0.02*
	USDA + NUU8	0.092 ± 0.03*	4.52 ± 0.02*	4.75 ± 0.02*

Values are the average of three replicates. ± values are standard deviations.

*Values significant at *p* 0.01. Soil nutrient contents were measured after 30 days of growth of the plant under greenhouse conditions.

### Analysis of soil enzyme activities

Data regarding soil enzymes showed that rhizobacteria treatments improved the protease, acid, and alkaline phosphomonoesterase activities in both conditions (Table 3). A single inoculation of *B. japonicum* USDA 110 significantly increased the protease, acid, and alkaline phosphomonoesterase compared to the control under both conditions. However, the co-inoculation of *B. japonicum* USDA 110 and *P. Putida* NUU8 significantly improved the activities of these enzymes under both conditions (Table 3). Co-inoculation of soybean with *B. japonicum* USDA 110 and *P. putida* NUU8 strains significantly enhanced protease activity by 19%, acid phosphomonoesterase activity by 10%, and acid phosphomonoesterase and alkaline phosphomonoesterase activity by 27% over the control under normal conditions. Co-inoculation with *B. japonicum* USDA 110 and *P. putida* NUU8 under drought stress conditions significantly increased the protease activity by 32%, acid phosphomonoesterase by 27%, and alkaline phosphomonoesterase by 19% over the control (Table 3).

**Table 3:** Effect of coinoculation with *B. japonicum* USDA 110 and *P. putida* NUU8 and single inoculation *B. japonicum* USDA 110 strains on soil enzymes under normal and drought conditions.

Conditions	Treatments	Protease activity (µg NH_4_^+^-N g^−1^ h^−1^)	Acid phosphomonoesterase activity (µg pNPg^−1^ h^−1^)	Alkaline phosphomonoesterase activity (µg pNPg^−1^ r^−1^)
Normal	Control	25.8 ± 0.08	725.1 ± 21.3	315.1 ± 10.1
	USDA 110	27.2 ± 0.12*	783.0 ± 22.4*	385.6 ± 16.4
	NUU8	24.2 ± 0.11	731.0 ± 19.3*	338.2 ± 13.4
	USDA + NUU8	30.6 ± 0.11*	799.6 ± 28.6*	399.2 ± 18.1*
Drought	Control	20.6 ± 0.05	683.2 ± 20.5	312.5 ± 11.2
	USDA 110	25.1 ± 0.07*	730.9 ± 23.1*	346.6 ± 17.3*
	NUU8	23.2 ± 0.06*	701.2 ± 21.2*	3176 ± 15.6*
	USDA + NUU8	27.3 ± 0.08*	750.4 ± 31.3*	372.2 ± 18.4*

Values are the average of three replicates. ± values are standard deviations.

*Values significant at *p* 0.01. Soil nutrient contents were measured after 30 days of growth of the plant under greenhouse conditions.

## Discussion

Drought stress has adverse effects on seed germination and growth in various plants. Several researchers^2,3,29^ reported a decrease in the germination rate in legumes crops by drought stress. The negative impacts of drought on seed germination, plant growth, nodulation, and soybean yield have been reported^2,3^. Mafakheri et al.^17^ reported a 73% decrease in soybean yield under drought stress conditions. PGPR strains like *Bradyrhizobium* sp. and *Pseudomonas* sp. improve drought tolerance and plant growth by modifying root architecture and the secretion of siderophore, phytohormones, and EPS^20,21,24^. Gholami et al.^50^ reported improved germination and growth in soybean due to the synergistic effect of co-inoculation of *B. japonicum* and *P. putida*. Inoculation with PGPR improves plant growth, development, nodulation, and yield of different crops^24,30,33,35^. Co-inoculation of *Rhizobium* sp. and other PGPR in bean and chickpea enhance nodulation, plant growth, and nutrient uptake^36,43^. Co-inoculation of *Rhizobium tropici* CIAT 899 and *P. polymyxa* DSM36 significantly increase plant growth and nodulation in common bean compared to inoculation with *Rhizobium* sp. alone under drought-stressed conditions^51^. Tewari and Arora^52^ reported a 50% increase in germination due to the inoculation with EPS producing *Pseudomonas aeruginosa* PF23 under stress. A wide variety of PGPR have been reported to produce EPS^53^, and they help crop plants in better root colonization^52^, better seed germination, and stress tolerance^55^. They enhance water retention by maintaining the diffusion of organic carbon sources^54^. Vardharajula et al.^56^ observed that *Bacillus* sp. synthesized osmolytes and antioxidants that facilitate plant growth under drought stress conditions. The synthesis of phytohormones by bacterial strains is another mechanism that imparts stress tolerance in plants^29,57^.

Drought stress also adversely affects plant nutrient uptake such as N, P, K, Ca, and Mg^20^. Several studies reported that drought stress reduces the concentration of N, K, and P in plant tissue and declines nutrient uptake from soil^58,59^. Drought stress is known to significantly decrease N content in cowpea^60^. He and Dijkstra^58^ reported that drought stress conditions significantly decline N and P in plant tissues. Results of the present study shows that co-inoculation with *B. japonicum* USDA 110 and *P. putida* strains NUU8 significantly increased the N content, P content, and K content compared to the control under drought conditions. PGPR is known to colonize the plant's rhizosphere, adhere to the root surface, and maintain moisture content^25,61–63^. This makes stable aggregates that help in nutrient absorption in plants^52^.

Drought stress exhibits adverse effects on soil nutrient availability, soil nutrient adsorption, and soil enzyme activities. Hinsinger et al.^64^ reported that drought-stressed conditions significantly decrease the soil nutrients such as N, P, K, and microelements such as B, Fe, Mn, and Zn. Drought-stressed in the soil is known to decrease enzyme activities^65^. The decrease in soil enzyme activities observed in this study is in agreement with the decrease in P available forms in the drought-stressed conditions^41^. The enhancement in soil enzymes such as protease, acid phosphomonoesterase, and alkaline phosphomonoesterase due to rhizobial inoculation has been observed by Fall et al.^66^ and Jabborova et al.^67^. Nitrogen fixing symbionts, alone or in combination with other rhizobacteria have been reported to improve growth, nutrient uptake and root architecture in soybean as well as to improve the resistance in soybean and other plants^46,47,68–76^.

## Conclusions

The application of PGPR exerts beneficial effects on plant growth and nodulation in soybean through increased uptake of nutrients such as N, P, and K in soil under normal and drought stress conditions. Inoculation with single strains of PGPR, i.e., *B. japonicum* USDA 110, improve soybean growth; however, co-inoculation of *B. japonicum* USDA 110 and *P. putida* NUU8 improves more growth, nutrient contents in soybean and soil, and activities of soil protease and acid and alkaline monophosphoeserase, as compared to the single inoculation and control under drought condition. Thus the combination of *B. japonicum* USDA 110 and *P. putida* NUU8 can serve as an effective and sustainable approach for improving the growth, nutrient contents, and enzyme activities in soybean and soil under drought-stressed conditions.

## Data Availability

Permissions were obtained to collect the Soybean (*Glycine max* L. Merr.) seeds from Leibniz Centre for Agricultural Landscape Research (ZALF), Müncheberg, Germany. Experimental research and field studies on plants were in accordance with the guidelines of ZALF, Müncheberg, Germany.
